# Conscious awareness is required for holistic face processing

**DOI:** 10.1016/j.concog.2014.05.004

**Published:** 2014-07

**Authors:** Vadim Axelrod, Geraint Rees

**Affiliations:** a The Gonda Multidisciplinary Brain Research Center, Bar Ilan University, Ramat Gan 52900, Israel; b UCL Institute of Cognitive Neuroscience, University College London, London WC1N 3AR, UK; c Wellcome Trust Centre for Neuroimaging, University College London, London WC1N 3AR, UK

**Keywords:** Holistic face processing, Unconscious processing, Visual awareness

## Abstract

Investigating the limits of unconscious processing is essential to understand the function of consciousness. Here, we explored whether holistic face processing, a mechanism believed to be important for face processing in general, can be accomplished unconsciously. Using a novel “eyes-face” stimulus we tested whether discrimination of pairs of eyes was influenced by the surrounding face context. While the eyes were fully visible, the faces that provided context could be rendered invisible through continuous flash suppression. Two experiments with three different sets of face stimuli and a subliminal learning procedure converged to show that invisible faces did not influence perception of visible eyes. In contrast, surrounding faces, when they were clearly visible, strongly influenced perception of the eyes. Thus, we conclude that conscious awareness might be a prerequisite for holistic face processing.

## Highlights

• We explore unconscious holistic face processing using composite face stimulus.
• We test influence of the invisible faces on judgments of the visible eyes.
• We use three different sets of face stimuli and subliminal learning procedure.
• We show that invisible faces did not influence perception of visible eyes.
• Conscious awareness might be a prerequisite for holistic face processing.

## 1 Introduction

Most people are good at recognizing faces, which is a capacity that is usually taken for granted. Yet, given that all faces are essentially very similar (e.g., all faces have a nose, eyes, and mouth; relative position of features is largely the same), the cognitive task of face recognition is far from straightforward. A key component of efficient facial processing is considered to be holistic processing (Farah, Wilson, Drain, & Tanaka, 1998; Richler, Cheung, & Gauthier, 2011) – the ability to perceive a face as a whole and not as a set of independent features. Probably the most spectacular demonstration of this phenomenon is the composite face effect (Young, Hellawell, & Hay, 1987) – that is, when a facial image is composed of the bottom and top halves of two different faces, where recognition of one half of a face (e.g., the top part) is modulated by that of the other half (e.g., the bottom part). Holistic face processing in general, and the composite face effect in particular, has been extensively explored in healthy populations using behavioral measures (for review: Rossion, 2013), functional MRI (e.g., Andrews, Davies-Thompson, Kingstone, & Young, 2010; Axelrod, 2010; Axelrod & Yovel, 2010, 2011; Schiltz, Dricot, Goebel, & Rossion, 2010; Schiltz & Rossion, 2006) and event-related potentials (e.g., Jacques & Rossion, 2009; Wiese, Kachel, & Schweinberger, 2013) as well as in participants with impaired face recognition (prosopagnosia) (Avidan, Tanzer, & Behrmann, 2011; Busigny & Rossion, 2011). However, whether conscious awareness is required for holistic face processing is not known.

Understanding the role of consciousness and conscious awareness is one of the fundamental challenges of the cognitive sciences (Baars, 1993; Dennett, 1993; Koch, 2004). Empirically, the level of conscious awareness is usually evaluated by an introspective, “subjective report” (whether a participant was aware of a stimulus) and “objective measure” (forced-choice discrimination, even for subjectively unaware stimulus) (Merikle & Daneman, 1998). While qualitative differences between conscious and unconscious perception have been debated for years (e.g., Cheesman & Merikle, 1986; Peremen & Lamy, 2014; Vorberg, Mattler, Heinecke, Schmidt, & Schwarzbach, 2003), numerous behavioral (e.g., Marcel, 1983; Mudrik, Breska, Lamy, & Deouell, 2011; Sklar et al., 2012) and neuroimaging (e.g., Axelrod, Bar, Rees, & Yovel, 2014; Dehaene et al., 2001; Fahrenfort et al., 2012; Sterzer, Haynes, & Rees, 2008) studies demonstrate that information can be processed unconsciously. Unconscious face processing is one of the widely explored types of unconscious processing. A large body of evidence suggests that emotional aspects of face processing (for reviews: Pessoa & Adolphs, 2010; Tamietto & de Gelder, 2010), gaze (Chen & Yeh, 2012; Stein, Peelen, & Sterzer, 2012; Stein, Senju, Peelen, & Sterzer, 2011) and face familiarity (de Gardelle, Charles, & Kouider, 2011; Henson, Mouchlianitis, Matthews, & Kouider, 2008; Kouider, Eger, Dolan, & Henson, 2009) can be processed without conscious awareness; however, several studies have shown that facial identity (Moradi, Koch, & Shimojo, 2005; Stein & Sterzer, 2011; Stone & Valentine, 2005) and face gender/race (Amihai, Deouell, & Bentin, 2011) cannot be processed unconsciously. In the present study, we addressed the question of unconscious face processing from another angle, while asking whether holistic face processing can take place unconsciously. Based on the previous negative results of unconscious face identity/gender processing, and given that face recognition and holistic processing might share common underlying mechanisms (Richler, Cheung et al., 2011; Wang, Li, Fang, Tian, & Liu, 2012), one possibility is that holistic face processing cannot be accomplished without conscious awareness. Alternatively, it is also possible that holistic face processing is a more basic type of processing than face recognition, which implies that holistic processing might still occur unconsciously. In addition, given that holistic processing has been suggested to be an automatic process (Richler, Wong, & Gauthier, 2011), it therefore possibly could be executed unconsciously (Hasher & Zacks, 1979).

**Fig. 1:**
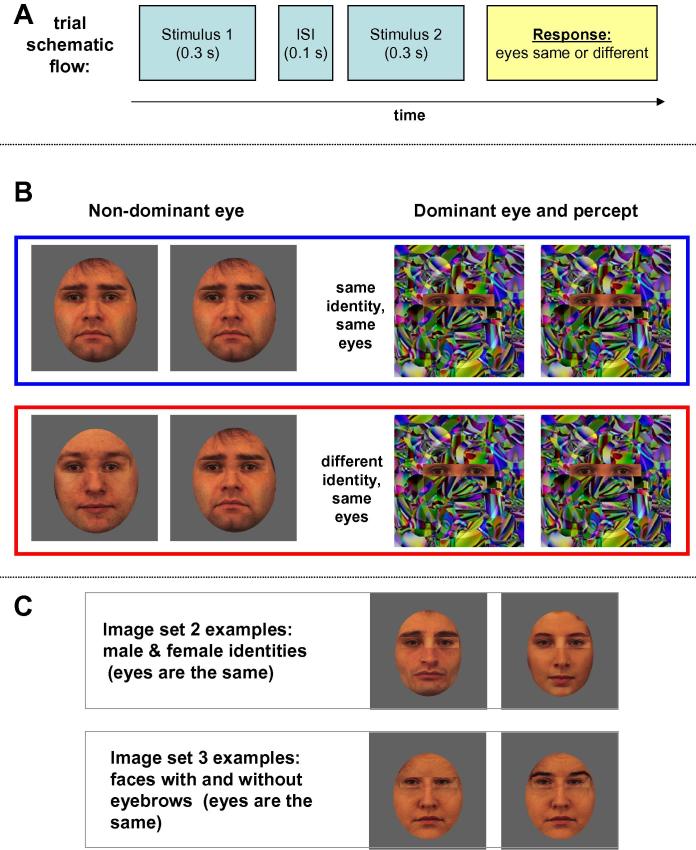
Experiment flow and examples of stimuli used in the study. (A) Schematic flow of a trial. (B) Examples of the stimuli while the left and right columns show the stimuli as they were projected to non-dominant and dominant eye, respectively. The right column also depicts the actual percept of the stimuli. Two rows (in red and blue frames) were the two conditions used in the experiment. Note that eyes were the same in all images. (C) Examples of the stimuli from the male and female image set (top) and from the image set “with and without eyebrows” (bottom).

In the present study, we devised a novel “eyes-face” composite stimulus that was composed of a pair of eyes plus the remaining part of the face [c.f., top and bottom image face parts (Young et al., 1987)]. Participants had to discriminate between pairs of eyes in two consecutively presented composite images while the rest of the face was either the same or different (Fig. 1A and B). Critically, in the subliminal version of the paradigm, while the eyes were always visible, the rest of the face was rendered invisible using Continuous Flash Suppression (CFS; Fig. 1B) (Harris, Schwarzkopf, Song, Bahrami, & Rees, 2011; Tsuchiya & Koch, 2005). We asked whether invisible faces influenced discrimination of the visible eyes.

## 2 Experiment 1

In this experiment, we used three different sets of composite faces. The first set was comprised of five male composite faces (examples of faces: Fig. 1B left side). To increase the perceptual differences between images, a second set included three male and three female composite faces (examples of faces: Fig. 1C, top). The third set of images was comprised of three female faces, either with or without eyebrows (examples of faces: Fig. 1C, bottom). The motivation to include this third composite image set was that eyebrows are the closest facial feature to the eyes and, consequently, have a higher chance of being attended to when the task is eyes discrimination.

Participants were presented with two consecutive composite faces that contained visible eyes and faces. The face part of each image was rendered invisible by CFS (Fig. 1B). The pairs of eyes in the two images were always the same, and the invisible faces were either the same or different. The task was to report whether successive pairs of visible eyes were the same or different. Participants were told that this eyes discrimination task was very difficult, and were encouraged to look out for the smallest differences between the two stimuli. Notably, because the stimuli did not appear in the exact same screen position (a small amount of spatial jitter was added; see Methods) and the eyes were surrounded by a constantly changing CFS mask, it was not evident that sequential eye stimuli were actually identical. Effect size was defined in percent units as the percent of trials answered “eyes same” when the invisible faces of the two images were the same minus the percent of trials answered “eyes same” when the invisible faces of the two images were different. An effect size larger than zero was taken as evidence of a subliminal influence of the invisible faces on judgments of the visible eyes.

### 2.1 Methods: Experiment 1

#### 2.1.1 Participants

Fifteen healthy volunteers (age 20–27 years, 10 females) participated in this experiment: all participants participated in the experiment with image set 1 (male faces), 14 of the same set of participants participated in the experiment with image set 2 (male and female faces), and 12 of the same set of participants took part in the experiment with image set 3 (faces with and without eyebrows). Two participants were excluded from the analysis of all three experiments because they reported that they could see the masked face. The experiment was approved by Tel-Aviv University ethics committee, and all participants gave informed consent to participate in the experiment.

#### 2.1.2 Apparatus

For stimuli presentation, a CRT 17-in. color monitor was used. Screen resolution was 1024 × 768, and refresh rate was 85 Hz. Stimuli were presented using MATLAB 7.6 with Psychtoolbox (Brainard, 1997). Participants sat in a comfortable chair at a distance from the monitor of 30 cm. During the experiment, the room lights were turned off.

#### 2.1.3 Stimuli

All image manipulations were performed in Adobe Photoshop CS2. Face stimuli (neutral face expressions) were taken from the Karolinska Directed Emotional Faces (Lundqvist & Litton, 1998). Image set 1 consisted of five male face identities (examples of faces: Fig. 1B, left side), and image set 2 consisted of three male and three female face identities (examples of faces: Fig. 1C, top). Image set 3 consisted of three female face identities – three original images and three images where the eyebrows were removed using Adobe Photoshop program (examples of faces: Fig. 1C, bottom). Five pairs of eyes (rectangle with the eyes, Fig. 1B) were cropped from different face images (not used in image sets 1–3) and integrated into each face image of the experimental image sets 1–3. The same pairs of eyes were used for all image sets. The size of the rectangle containing the eyes was identical for all pairs of eyes (see below). The resultant image sets included 25 stimuli each (five face identities with five pairs of eyes) for image set 1, and 30 stimuli each (six face identities with five pairs of the eyes) for image sets 2 and 3. None of the composite faces had original (“native”) eyes. The images were in color. Face images were positioned in the center of the rectangular background image frame, which was a light grey color (RGB: 97, 97, 97) (see Fig. 1B). Dimensions of the stimuli were as follows (in degrees of visual angle): background square frame (vertical: 27, horizontal: 27); face image (vertical: 21, horizontal: 16.5); eyes rectangle (vertical: 3.3, horizontal: 14.5).

#### 2.1.4 Invisibility manipulation

To render stimuli invisible, we used Continuous Flash Suppression (CFS) (Tsuchiya & Koch, 2005). The paradigm was designed in such a way that the eyes rectangle was fully visible with both eyes, whereas the remaining part of the face was invisible (Harris et al., 2011). During the experiment, participants wore cardboard anaglyph red/cyan glasses. The visible part of the stimulus was projected using all three RGB colors. The invisible part of the stimulus was projected using the red color channel (visible using red filter), while a Mondrian mask was projected using green/blue channels. The Mondrian mask appeared over the whole background rectangle frame with the exception of the visible stimulus part (see Fig. 1B). The position of the visible eyes rectangle was fixed relative to the Mondrian image. In addition, on the Mondrian masks, we drew an elliptic contour line at the location of the invisible face (see Fig. 1B, right side). The motivation for adding this contour ellipse was to urge participants to make eye judgments as though the eyes were part of a face. The elliptic contour line was created by increasing the brightness of the corresponding mask pixels by 20%. The ellipse contour was created at the same position for all mask images and was at a fixed position relative to the eyes rectangle. Mondrian masks were prepared by randomly scrambling a kaleidoscope image. Our preliminary pilots showed that use of this pattern achieved higher invisibility effects than the geometrical shapes usually used (e.g., Tsuchiya & Koch, 2005).

Mondrian masks were replaced continuously at a frequency of 10 Hz (every 100 ms). During the invisible part of Experiment 1, the face composite stimuli were always projected to the non-dominant eye of the participant while the CFS mask was presented to the dominant eye. Eye dominance was tested by asking participants to view a distant object through a hole made by the fingers of their two hands (“Miles test”) (Mendola & Conner, 2007; Miles, 1930). In the visible sessions, the participants wore specially prepared glasses with two red lenses (stimuli visible through both eyes and mask invisible through both eyes). Using this approach, we preserved the same quality of stimulation for visible and invisible sessions.

#### 2.1.5 Experimental design

For each one of three image sets, there were three experimental sessions: eyes discrimination session with invisible face images, awareness invisibility test of discriminating invisible face images, and eyes discrimination session with visible face images. To minimize the number of switches between tasks, participants first performed three sessions of eyes discrimination with invisible face images (all three image sets), then three sessions of awareness invisibility (all three image sets; explained below), and finally three sessions of eyes discrimination with visible face images (all three image sets). The order of the image sets within these three sessions was counterbalanced across participants. From the side of stimuli presentation (computer code), all three experimental sessions were exactly the same for each image set.

A trial consisted of two consecutively presented stimuli (each stimulus duration = 0.3 s, interstimulus interval [ISI] = 0.1 s) (Fig. 1A). There was a random position jitter between two stimuli (2% of stimulus size). The eyes in two images of a trial were always the same, whereas the faces were either the same (50% of the trials) or different (50% of the trials). For image set 1 (males only set), the different faces were of different male face identities. For image set 2 (males and females set) the different faces were each composed of different identities from opposite genders (either male–female or female–male, counterbalanced). For image set 3 (with and without eyebrows), the different faces were the same identity presented twice with and without eyebrows (either no eyebrows – eyebrows or eyebrows – no eyebrows). In the “invisible” sessions, only the eyes were visible; in the “visible” sessions (at the end of the experiment), both the eyes and faces were visible. Participants were asked to make their response after the second stimulus disappeared.

In the eyes discrimination experiment, participants had to press ‘1’ if the two pairs of eyes were the same and ‘2’ if the two pairs were different. The fact that the pairs of eyes were always the same was not known by participants. Participants were told before the experiment that the task would be very difficult and that any slight difference that they perceived between the two eye pairs should be taken as an indication of the presence of a difference between the eyes. Our preliminary pilot tests showed that presenting the two stimuli with a slight spatial jitter and a constant change in CFS mask around the eyes created an impression that the eyes were not actually identical. Indeed, at the informal debriefing after the experiment, participants indicated that they had seen differences between the pairs of eyes.

Awareness test sessions were very similar to the eyes discrimination sessions; the only difference was that participants had to discriminate between invisible faces (‘1’: same faces, ‘2’: different faces); since the participants admitted seeing nothing, they were encouraged to guess. Participants were asked not to use the visible eyes in the discrimination; instead, they were encouraged to try to discriminate based on the non-eye part of the image (inside the ellipse). Each session consisted of 50 trials (25 trials with the same faces and 25 with different faces). At the beginning of the experiment, before the first session with invisible face images, participants underwent a short training session (10 trials) of eyes discrimination with invisible faces. As face stimuli, we used two male identities that were not used afterwards in the experimental sessions.

#### 2.1.6 Data analysis

Data were analyzed using MATLAB and SPSS 17 software. The effect size for the eyes discrimination task was calculated as the percent of trials answered “same” when the face identities of the two images were actually the same minus the percent of trials answered “same” when the face identities were different. An effect size larger than zero provided evidence that a change in face influenced perception of the eyes. Significance at the group level was established using non-parametric Wilcoxon Signed Rank test (signrank MATLAB function). In the visual awareness tests, the individual *d*-prime values [signal detection theory (Macmillan, 2002)] of discrimination invisible stimuli (correct/incorrect) were assessed using non-parametric Wilcoxon Signed Rank test vs. zero. For all the parametric tests (ANOVAs and correlations), the data was first tested for normality using Lilliefors test (lillietest MATLAB function). Reaction times of participants were not analyzed since the participants were asked to respond only when the second stimulus of the trial disappeared, and they were asked to maximize accuracy and not to minimize response time. To calculate the Bayes factor, we used the online calculator of Zoltan Dienes (http://www.lifesci.sussex.ac.uk/home/Zoltan_Dienes/inference/Bayes.htm).

### 2.2 Results: Experiment 1

**Fig. 2:**
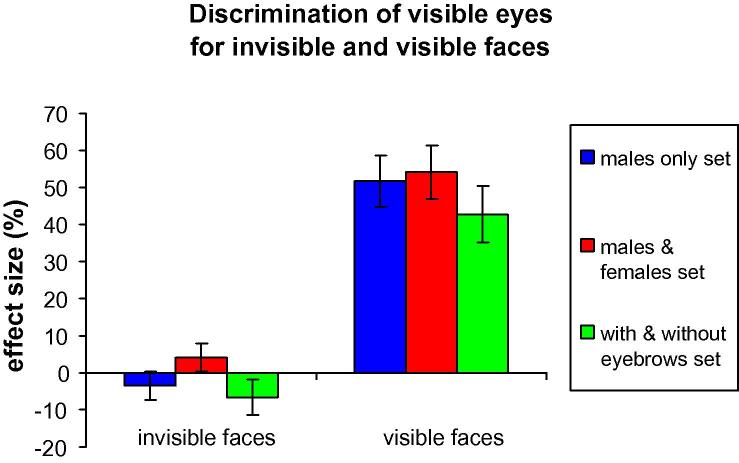
Results of Experiment 1: discrimination of the visible eyes for invisible and visible faces (three image sets). Error bars denote standard error of the mean.

Judgment of visible eyes was not influenced by invisible faces in any of the image sets (Fig. 2, left side) [males only set 1: effect size: −3.4%, MSE: 3.7%, *p* = .26, Wilcoxon sign-rank = 29.5, one Sample Wilcoxon Signed Rank test vs. 0; males and females set 2: effect size: 4.1%, MSE: 3.7%, *p* = .29, Wilcoxon sign-rank = 27; with and without eyebrows set 3: effect size: −6.4%, MSE: 4.8%, *p* = .19, Wilcoxon sign-rank = 11.5]. For raw response rates of “same eyes” answers, see Table 1, first row. Invisibility of faces was tested in separate sessions, with stimulus configurations identical to the main experiment, but the participants were required to discriminate between the invisible faces. Discrimination of faces did not differ significantly from chance for all three image sets [males only set 1: *d*-prime: 0.082, MSE: 0.11, *p* = .47, Wilcoxon sign-rank = 25; males and females set 2: *d*-prime: 0.15, MSE: 0.2, *p* = .50, Wilcoxon sign-rank = 25.5; males and females set 3: *d*-prime: 0.028, MSE: 0.13, *p* = .95, Wilcoxon sign-rank = 22] (raw response rates, Table 1, second row). Contrarily to invisible faces, judgment of visible eyes was strongly influenced by visible faces in all image sets (Fig. 2, right side) [males only set 1: effect size: 51.7%, MSE: 7%, *p* = .001, Wilcoxon sign-rank = 0; males and females set 2: effect size: 54.2%, MSE: 7.2%, *p* = .002, Wilcoxon sign-rank = 0; with and without eyebrows set 3: effect size: 42.8%, MSE: 7.6%, *p* = .005, Wilcoxon sign-rank = 0] (raw response rates, Table 1, third row). The difference between invisible and visible faces was confirmed by the two-way repeated measured ANOVA analysis with image set and face visibility as factors: there was a highly significant main effect of face visibility: *F*(1, 9) = 118.425, *p* < .001 but non-significant main effect of image set [*F*(2, 18) = 3, *p* = .075] and non-significant interaction between image set and face visibility [*F*(2, 18) < 1].

Experiment 1 found no evidence that invisible faces influence the perception of visible eyes. Notably, the “null effect” for invisible faces may stem either from a lack of sensitivity or, alternatively, from the phenomenological absence of unconscious processing (Dienes, 2011, in press). Since the orthodox (Neyman and Pearson) statistical approach is unable to distinguish between these two possibilities, we adopted a Bayesian approach (Dienes, 2011). That is, the Neyman and Pearson approach, by definition, might only find support for the alternative hypothesis (rejecting H_0_ and accepting H_1_), but cannot provide support for the H_0_ hypothesis (when H_1_ is not accepted). A Bayesian approach (Bayes factor), in contrast, can provide support for either H_0_ or H_1_ (Dienes, in press). As the data input parameters, we used effect size and mean square error (Jiang et al., 2012). To model the expectation parameters, following the recommendation of Dienes (Dienes, 2011), we consulted previous studies that also explored unconscious face processing using the CFS paradigm (Amihai et al., 2011; Moradi et al., 2005; Yang, Hong, & Blake, 2010). In particular, the effect size during unconscious face processing in these studies was at least two times smaller compared to that during conscious processing (Yang et al., 2010), whereas in some cases it was five to ten times smaller (Amihai et al., 2011; Moradi et al., 2005). Therefore, given that the effect size in the conscious condition of our study was on average 50%, the unconscious mean prediction was defined as 7.5% and the standard deviation was set to 5% (two-tailed normal distribution). The results of the analysis revealed the following: for the males-only set 1, the Bayes factor was 0.07; for the males-and-females set 2, the Bayes factor was 0.95; and for the eyebrows set 3, the Bayes factor was 0.23. Thus, the Bayes factor values in sets 1 and 3 provide strong support for the phenomenological absence of unconscious processing (Bayes factor < 0.33); the results of set 2 should be interpreted as a “lack of sensitivity” (0.33 < Bayes factor < 3) (Dienes, 2011).

## 3 Experiment 2

**Fig. 3:**
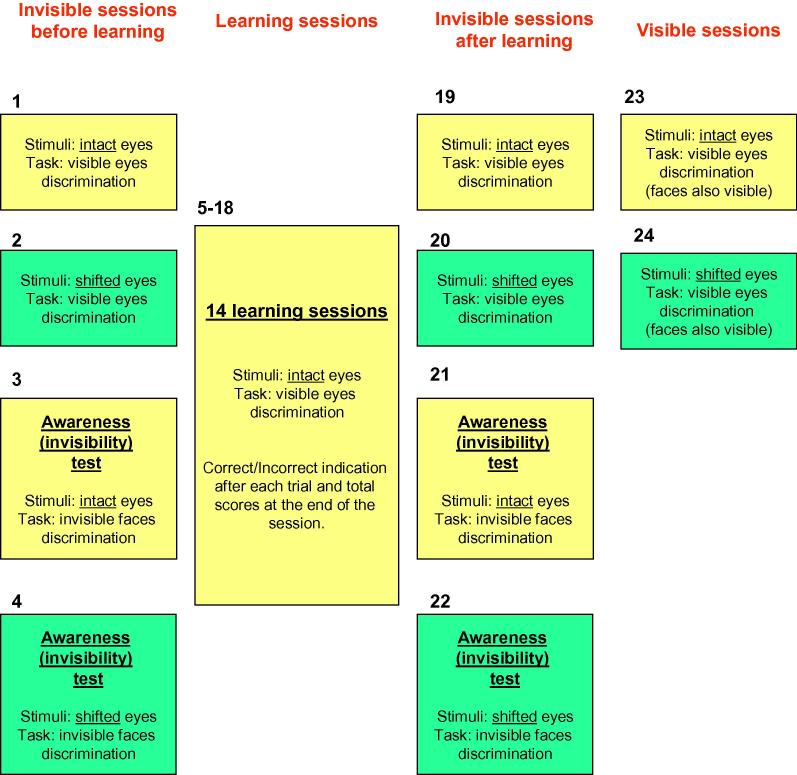
Schematic flow of Experiment 2. color coding stands for different types of stimuli (yellow: intact eyes; cyan: shifted eyes). A number near the rectangle denotes session ID from the start of the experiment. The order of sessions 1–2, 3–4, 19–20, 21–22, and 23–24 was counterbalanced across participants. (For interpretation of the references to color in this figure legend, the reader is referred to the web version of this article.)

**Fig. 4:**
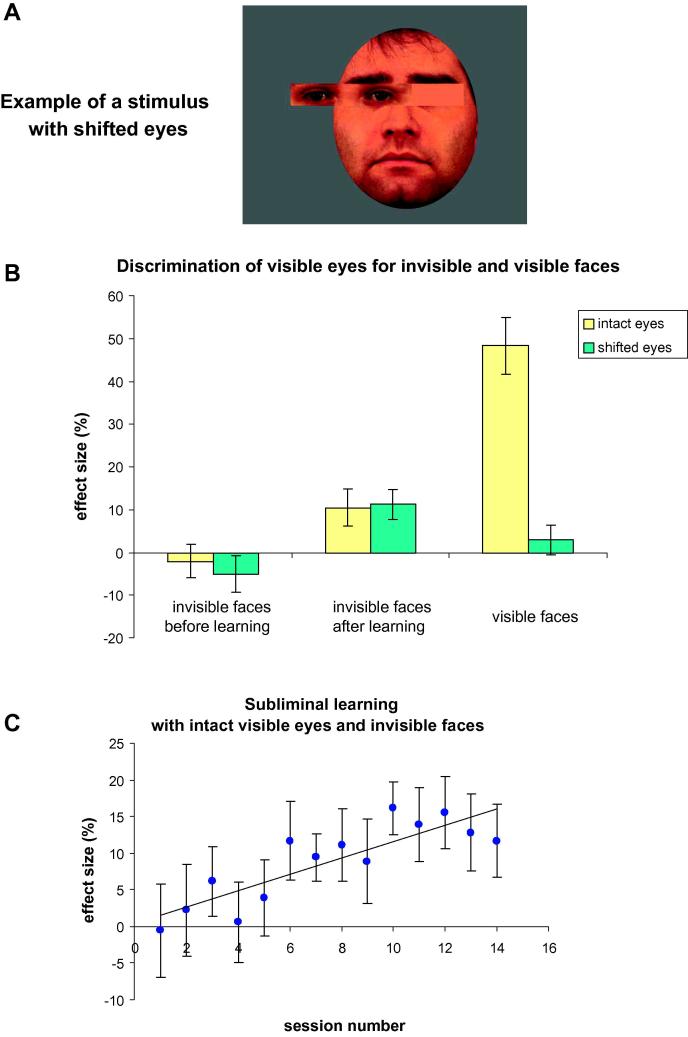
Example stimulus and the results of Experiment 2. (A) Example of a stimulus with shifted eyes. The stimuli were in color (as in Fig. 1). (B) Results of discrimination of the visible eyes for invisible (before and after learning) and visible faces. Error bars denote standard error of the mean. (C) Across participants results of subliminal learning for 14 sessions. Line denotes a linear regression. Error bars denote standard error of the mean.

In the current experiment, we asked whether there was a way to improve unconscious holistic processing by means of subliminal learning with feedback (e.g., Atas, Faivre, Timmermans, Cleeremans, & Kouider, 2014; Di Luca, Ernst, & Backus, 2010; Nishina, Seitz, Kawato, & Watanabe, 2007; Rosenthal & Humphreys, 2010; Watanabe, Náñez, & Sasaki, 2001). Participants underwent half an hour of subliminal learning with feedback, which was based on same/different invisible faces (see Methods). We hypothesized that by means of learning, we could induce the invisible face to modulate perception (discrimination) of the visible eyes. The flow of this experiment is shown in Fig. 3. This experiment used the image set with five male faces (two versions: intact and shifted eyes; described below). To verify that, as a result of repetitive exposure during learning, the invisible part of the stimulus (face) did not become visible (e.g., Atas, Vermeiren, & Cleeremans, 2013; Schwiedrzik, Singer, & Melloni, 2009, 2011), participants underwent an invisible face awareness test before and after learning. In addition, unrelated to subliminal learning, participants were tested with a set of stimuli in which the original five male stimuli were manipulated in such a way that the rectangle of the eyes in all images was shifted (Fig. 4A). Similar manipulations are effective in disrupting holistic face processing (e.g., Axelrod & Yovel, 2010; Maurer, Grand, & Mondloch, 2002; McKone, Kanwisher, & Duchaine, 2007). Our plan was to compare the magnitude of any influence of invisible faces between an intact and shifted eyes stimuli set. Critically, in order to conclude that any effect, if found, was related to holistic processing, the magnitude of this effect must be higher for the intact eyes than for the shifted eyes stimuli set.

### 3.1 Methods: Experiment 2

#### 3.1.1 Participants

Eighteen healthy volunteers (ages 19–30, 13 females) participated in this experiment. Two participants were excluded from the analysis because they reported consciously seeing the masked face. The experiment was approved by Tel-Aviv University’s ethics committee, and all participants gave informed consent to participate.

#### 3.1.2 Apparatus

The same as in Experiment 1.

#### 3.1.3 Stimuli

The image set with five male identities from Experiment 1 was used in this experiment. In addition, based on this image set, we created a second image set where the eyes rectangle was shifted to the left 6.5 degrees of a visual angle (see Fig. 4A). The empty eye position was filled with a uniform average color taken from the surrounding facial features (RGB: 217, 112, 67). The horizontal size of the background rectangle frames for both intact and shifted eye sets were increased to 35° of a visual angle.

#### 3.1.4 Invisibility manipulation

The same as in Experiment 1.

#### 3.1.5 Experimental design

The flow of the experiment is presented in Fig. 3. For each of the two image sets (intact and shifted eyes), there were five experimental sessions: two sessions with invisible face images, where eyes had to be discriminated (one before and one after learning), two awareness invisibility tests of discriminating invisible face images (one before and one after learning), and an eyes discrimination session with visible face images. The awareness invisibility tests always followed the eyes discrimination sessions with invisible face images. The eyes discrimination session with visible face images was always the last session of the experiment. The order of intact and shifted eyes sessions was counterbalanced between participants. All sessions of this experiment (including the learning sessions; see below) consisted of 30 trials (15 trials with same faces and 15 with different faces).

The learning procedure included 14 sessions. These learning sessions had the same design as the testing sessions (eyes discrimination test) with the exception of a correct/incorrect indication after each trial (feedback) and overall score at the end of the session. While participants’ task was to discriminate between pairs of eyes, the correct/incorrect answer indication was based on same/different invisible faces. In particular, the answer was defined as correct if the participant answered “same” when the two invisible identities were the same or answered “different” when the two invisible identities were different. At the end of each learning session, participants received their session scores (percentage of correct answers). At the informal debriefing after the experiment, participants were asked based which parameters had served as the basis for their development of the ability to discriminate between the pairs of eyes. All participants indicated that their decision had been based on either eye shape, distance between the eyes, or eye color.

#### 3.1.6 Data analysis

The analysis procedure of non-learning sessions was the same as in Experiment 1. The significance of a learning effect was evaluated using linear regression analyses, which were applied for group (averaged) and individual data. The procedure for the group-level linear regression analysis was as follows: (1) effect size values for each session were averaged across participants resulting in averaged effect size values (Fig. 4C); (2) these effect size values were submitted to linear regression; and (3) a regression line slope coefficient significantly different from zero was used to support the learning effect (Weisberg, 2005). The procedure for the individual-level linear regression analysis was as follows: (1) for each participant, the effect size values were submitted to linear regression; (2) the individual linear regression slope coefficients were obtained and submitted to Wilcoxon Signed Rank test (slope coefficients vs. zero test).

### 3.2 Results: Experiment 2

In line with the results of Experiment 1, before learning, invisible faces did not influence the judgment of visible eyes for both intact and shifted eye sets (Fig. 4B, leftmost bars) (intact eyes: effect size: −2.1%, MSE: 3.9%, *p* = .69, Wilcoxon sign-rank = 60.5; shifted eyes: effect size: −5%, MSE: 4.3%, *p* = .25, Wilcoxon sign-rank = 29) (raw response rates, Table 2, first row). The Bayes factor, modeled with the same parameters as in Experiment 1, was 0.23 for the intact eyes set and 0.21 for the shifted eye set. Thus, for both image sets, the results of the Bayesian analysis (Bayes factor) suggested the absence of unconscious processing.

Participants then completed a subliminal learning task with feedback using the intact eyes image set. Average learning results across participants are shown in Fig. 4C; as seen, performance gradually improved across sessions (slope coefficient = 1.12, significantly different from zero: *t*(13) = 5.56, *p* < .001). To examine the effect of learning at an individual level, a linear regression model was estimated for each participant; the resultant individual slope coefficients were significantly above zero [Wilcoxon Signed Rank test: *p* = .0086, Wilcoxon sign-rank = 5.5]. After learning, participants were tested again (sessions without feedback), and we observed a significant effect of the invisible face on visible eyes judgments for both intact and shifted eyes sets (Fig. 4B, middle bars) [intact eyes: effect size: 10.4%, MSE: 4.3%, *p* = .029, Wilcoxon sign-rank = 26; shifted eyes: effect size: 11.2%, MSE: 3.4%, *p* = .013, Wilcoxon sign-rank = 7.5] (raw response rates, Table 2, second row). Repeated-measures ANOVA, with eye position (intact/shifted eyes) and time of test (before/after learning) as factors, revealed a highly significant main effect of time of test [*F*(1, 15) = 11.826, *p* = .004]; however, no significant effect of eye position [*F*(1, 15) < 1] and no significant interaction [*F*(1, 15) < 1] were found, which confirms the similar effect size for intact and shifted eyes. In addition, using the Bayesian approach, we tested whether the absence of difference between unconscious processing for intact and shifted eyes should be interpreted as an insufficient experimental sensitivity or as positive evidence in support of the absence of unconscious holistic processing. The data mean value was the difference in effect size between intact and shifted eyes (equal to 0.83%), and the mean standard error of difference was 0.7%. The modeling parameters were estimated based on the reduction of the effect for unconscious processing compared to conscious processing (as in Experiment 1) and the effect size for intact vs. shifted visible faces (around 50%). Thus, the model’s mean was set to 5% and the standard deviation was set to 5% (two-tailed normal distribution). The Bayes factor we found was 0.16, unequivocally suggesting the absence of unconscious holistic processing.

Interestingly, as can be seen in Table 2 (second vs. first raw), the learning effect was associated with an increase in the proportion of “same” responses for the “same” invisible faces (but no major change for “different” invisible faces). To test this statistically, for raw responses (“same” responses), we ran three-way repeated-measures ANOVA with eyes position (intact/shifted eyes), time of test (before/after learning) and invisible stimulus type (same/different faces) as factors. We found significant two-way interaction between time of test and invisible stimulus type *F*(1, 15) = 11.826, *p* = .004]. The follow-up repeated-measures ANOVA only for “same” invisible faces with eyes position (intact/shifted eyes) and time of test (before/after learning) as factors revealed a significant main effect of time of test [*F*(1, 15) = 6.025, *p* = .027], no significant effect of eye position [*F*(1, 15) = 2.596, *p* = .128] and no significant interaction [*F*(1, 15) < 1]. Similar repeated-measures ANOVA only for “different” invisible faces revealed no significant effects (insignificant main effect of time of test [*F*(1, 15) < 1], eye position [*F*(1, 15) = 2.163, *p* = .162] and interaction between them [*F*(1, 15) < 1]). Thus, we conclude that the learning was associated with increasing the number of “same” responses for “same” invisible faces, but no change for “different” invisible faces.

To ensure that faces were genuinely invisible, we ran awareness tests before and after learning (Fig. 3). These awareness tests confirmed that the faces were invisible before [intact eyes: *d*-prime: 0, MSE: 0.1, *p* = .84, Wilcoxon sign-rank = 36.5; shifted eyes: *d*-prime: 0.023, MSE: 0.1, *p* = .97, Wilcoxon sign-rank = 45] and after [intact eyes: *d*-prime: 0.069, MSE: 0.096, *p* = .43, Wilcoxon sign-rank = 46; shifted eyes: *d*-prime: 0.097, MSE: 0.12, *p* = .39, Wilcoxon sign-rank = 45] training. For both types of eyes, no differences existed in visibility level before or after learning [intact eyes: *p* = .63, Wilcoxon sign-rank = 51.5; shifted eyes: *p* = .68, Wilcoxon sign-rank = 60] and no significant correlation existed across participants between the level of face discrimination (awareness test) and level of eyes influence effect (eye judgment main task) [intact eyes: *r*(15) = 0.15, *p* = .56; shifted eyes: *r*(15) = 0.28, *p* = .28]. Taken together, these results suggest that because of learning, participants’ judgment of eyes was influenced by invisible faces. Critically, because the results for intact and shifted eyes were similar, we conclude that the effect is not related to unconscious holistic face processing [the classical composite face effect (Young et al., 1987)].

Finally, we tested the “eyes-face” stimulus but now with visible faces (Fig. 4B, rightmost bars). The findings revealed that, while in the intact eyes condition, faces influenced the perception of the eyes significantly [effect size: 48.3%, MSE: 6.6%, *p* < .001, Wilcoxon sign-rank = 1.5], the effect was completely abolished for the shifted eyes [effect size: 2.9%, MSE: 3.5%, *p* = .44, Wilcoxon sign-rank = 34.5]. This finding confirms that the shifted eyes manipulation disrupted holistic face processing. To examine the difference between visible and invisible perception (after learning), we ran a two-way repeated-measures ANOVA with visibility level (visible/invisible) and eye position (intact/shifted) as factors. The results showed a significant main effect of visibility level [*F*(1, 15) = 11.7, *p* = .004], a significant main effect of eye position [*F*(1, 15) = 26.5, *p* < .001], and a significant interaction [*F*(1, 15) = 22.9, *p* < .001]. To explore different patterns of visible and invisible processing further, we ran a post hoc non-parametric Wilcoxon Signed Rank test, which revealed a larger effect size for visible compared to invisible for intact eyes [*p* < .0012, Wilcoxon sign-rank = 5.5] and a trend for a larger effect size for invisible compared to visible for shifted eyes [*p* < .077, Wilcoxon sign-rank = 24.5].

## 4 Discussion

The objective of the current study was to test whether holistic face processing can occur outside of conscious awareness. We used a novel “eyes-face” stimulus, where discrimination between sets of eyes was affected by the presentation of a visible congruent or incongruent face. Using different sets of composite face stimuli, we showed that visible but not invisible faces influenced perception of visible eyes. Moreover, even after subliminal learning, when invisible faces biased judgments of visible eyes, this effect was not found to be related to holistic face processing. Thus, we conclude, conscious awareness may be necessary for holistic face processing.

The question of whether face recognition and face identity-related processing can be accomplished unconsciously has been at the focus of research in recent years. In particular, several studies have shown that neither facial identity (Moradi et al., 2005; Stein & Sterzer, 2011; Stone & Valentine, 2005) nor face gender/race (Amihai et al., 2011) is processed unconsciously. In the current study, we hypothesized that, if holistic processing is a more basic component of face recognition, then it might be possible to find evidence for unconscious holistic face processing. To increase the chances of identifying this effect, several steps were taken. First, the “eyes-face” stimulus used was optimized to generate a strong effect for visible faces. That is, studies with a classical composite face illusion stimulus (top and bottom face halves) often use response time to index holistic processing because response accuracy measures are often not sensitive enough (e.g., de Heering & Rossion, 2008; Wang, Li et al., 2012). Here, given the strong response accuracy effect for visible faces (Fig. 4B), relatively large amount of room is left for a potential effect with invisible faces. Second, in Experiment 1, we used different sets of faces while aiming to maximize perceptual differences between the faces (e.g., male and female faces). Third, by using the image set in Experiment 1 with and without eyebrows, we ensured that the facial features (eyebrows) which were essential for inducing the effect were as close as possible to the visible eyes rectangle. Finally, we employed a subliminal learning procedure, which was successful in the sense that, as a result of learning, invisible faces influence responses regarding visible eyes (discrimination of visible eyes). However, the fact that similar effects were found for intact and shifted eyes ruled out the interpretation that the effect was related to holistic face processing. Thus, given that despite all aforementioned steps, no unconscious holistic face processing effect could be found, we suggest that this process might not be processed unconsciously, at least for the condition of dichoptic stimulation employed here.

The absence of holistic face processing outside conscious awareness is also interesting to consider in light of the recent proposal that the holistic phenomenon, as it is measured in a composite task (e.g., Young et al., 1987), is a result of automatic processing with a failure to allocate covert attention (Richler, Cheung et al., 2011). Accordingly, given that some define automatic processes as unconscious (e.g., Hasher & Zacks, 1979), one could have expected that face holistic processing might be executed unconsciously. Several lines of evidence can reconcile our result with these expectations. First, the link between automatic and unconscious processing is frequently made for the highly learned processes, like car driving (e.g., Charlton & Starkey, 2011), which might involve awareness mechanisms different from the sensory (un)awareness for masked stimuli used in our study. Second, the relationship between attention and consciousness is highly debated (for reivews: Koch & Tsuchiya, 2007; Marchetti, 2012; Van Boxtel, Tsuchiya, & Koch, 2010) and it has been shown, for example, that two processes can be dissociated (e.g., Naccache, Blandin, & Dehaene, 2002). Yet, it is not clear whether a failure to allocate covert attention, which might be a result of automatic processing (Richler, Cheung et al., 2011), can occur for invisible, unconscious stimuli. Finally, a similar hypothesis had been proposed with regard to the Stroop task (Stroop, 1935), which is also automatic (MacLeod, 1991) and therefore can be processed unconsciously (Marcel, 1983). Yet, the empirical support for this hypothesis is rather controversial: while some studies do find an unconscious Stroop effect (e.g., Marcel, 1983), others claim that such an effect can be explained by conscious awareness (Tzelgov, Porat, & Henik, 1997) or partial awareness (Kouider & Dupoux, 2004). Thus, in light of the evidence provided, the absence of unconscious holistic face processing might not be that surprising.

The results reported here are interesting to consider in the context of unconscious processing of visual context in general. The invisible surroundings can influence the orientation of a centrally presented visible grating (Clifford & Harris, 2005), and the invisible surrounding luminance can modulate the perceived brightness of the centrally presented visible circle (Harris et al., 2011). In addition, two studies have explored the perception of illusory contours surrounded by an invisible context, and reported mixed results. One study found, using breaking continuous flash suppression (Jiang, Costello, & He, 2007) that an invisible Kanizsa triangle emerged into awareness faster than did a control stimulus (Wang, Weng, & He, 2012). The second asked participants to indicate the direction of the Kanizsa triangle induced by the invisible surroundings and reported only chance performance (Harris et al., 2011). Notably, the invisible context and the stimuli explored in the studies described above were relatively simple stimuli, which are known to be processed at a relatively low level within the visual hierarchy (e.g., V1 for line orientation, V4 for color processing). In contrast, faces are processed within high-level regions of the occipito-temporal cortex (Haxby, Hoffman, & Gobbini, 2000); therefore, it is plausible that low-level, but not high-level, context can be processed unconsciously. Finally, Mudrik and colleagues recently demonstrated an interesting example of invisible context processing (Mudrik et al., 2011), where the authors showed that invisible scenes containing incongruent invisible objects emerged into awareness faster than did congruent scenes (see also: Mudrik & Koch, 2013). Yet, as the invisible context in this study was semantic and not purely visual, there is no straightforward way to relate their findings to ours.

What might be the possible underlying neural mechanisms of the effect we observed? The neural network of face processing is well characterized (Haxby et al., 2000), while the commonest and the most reproducible region across participants is the Fusiform Face Area (FFA) (Kanwisher, McDermott, & Chun, 1997). The FFA exhibits various properties pertinent to face processing, such as partial view-invariance (e.g., Axelrod & Yovel, 2012; Grill-Spector et al., 1999; Kietzmann, Swisher, König, & Tong, 2012) and discrimination between face identities (e.g., Gilaie-Dotan & Malach, 2007; Nestor, Plaut, & Behrmann, 2011; Rotshtein, Henson, Treves, Driver, & Dolan, 2005). More critical for the current discussion, however, is that the FFA has been suggested to be responsible for holistic face processing (Andrews et al., 2010; Axelrod & Yovel, 2010, 2011; Schiltz & Rossion, 2006; Zhang, Li, Song, & Liu, 2012). If this is indeed the case, and assuming that unconscious information can reach the FFA (e.g., Fahrenfort et al., 2012; Moutoussis & Zeki, 2002; Sterzer et al., 2008), then our result suggests that the FFA operations responsible for holistic processing are associated with awareness; however, one should also consider that the network of face-processing regions is distributed (Gobbini & Haxby, 2007) and spans not only the occipito-temporal cortex (Op de Beeck, Haushofer, & Kanwisher, 2008) but also the frontal lobes (Axelrod & Yovel, 2013; Ishai, Schmidt, & Boesiger, 2005). As such, holistic processing might also require interactive processing between several different brain regions – the type of distributed, long-range processing that was proposed to be associated with awareness (e.g., Dehaene & Changeux, 2011; Dehaene, Kerszberg, & Changeux, 1998). Overall, future research will be needed to gain a deeper understanding of the underlying neural correlates.

A special note should be made regarding the subliminal learning procedure employed here. As discussed, the fact that, during and after learning, invisible faces influenced both intact and shifted eyes suggests that the effect was not related to holistic processing. We also found that the learning was associated with a more frequent “same” answer for the “same” invisible faces, but no change for “different” invisible faces. Yet, as the goal of the current study was not to explore the mechanisms of subliminal learning but rather to use this type of learning as a tool, it is not possible to clearly determine exactly what type of information was learnt and how the learning occurred. It is plausible that, in the course of learning, participants unconsciously learnt to associate between the “change” or “no change” of invisible images (faces) and the required response for visible eyes. In other words, the same/different faces were treated as just same/different images. In addition, it is also possible that motor response mapping associative learning occurred (Damian, 2001) while participants learnt to press ‘1’ when two invisible images were the same and ‘2’ when they were different. Notably, the learning procedure did not influence face awareness, which was equally invisible before and after learning.

Finally, it should be noted that according to an alternative view of holistic processing, the effect that is measured by a composite task might not be of a perceptual but rather a decisional nature (e.g., Richler, Gauthier, Wenger, & Palmeri, 2008; Richler, Palmeri, & Gauthier, 2012; Rossion, 2013). In our study, to increase the sensitivity of the design, we included only the pairs of the same eyes. That is, by requiring participants to discriminate between the same eyes, we imposed the participants to set the same-different decision boundary at a low level. Indeed, this design was very sensitive, as we found that context had large effects on eye perception for visible faces. Yet, this design had its downside as well; since no different pairs of eyes were included, our ability to estimate potential response bias was limited (e.g., Richler et al., 2012; Rossion, 2013). Critically, the main goal of the present study was to explore whether invisible faces influence the discrimination of visible eyes, regardless of the nature of holistic processing.

To conclude, in the current study we explored the question of whether holistic processing can take place outside of conscious awareness. Using three different sets of face images and applying a procedure of subliminal learning, we demonstrated that conscious visual awareness might be a prerequisite for holistic processing.

## Figures and Tables

**Table 1:** Experiment 1: percent of trials answered the “same” when faces were “same” or “different”. In the other words, the values denote the percentages of the same answers binned for the type of stimuli (same or different faces). Columns represent three image sets and rows represent task and visibility manipulation. Values in parentheses denote standard error of the mean.

	Males only set 1	Males and females set 2	Eyebrows set 3
Invisible faces	Same: 62.1% (5.4%)	Same: 71.6% (4.6%)	Same: 65.2% (4.5%)
Task: eyes discrimination	Different: 65.5% (4.8%)	Different: 67.5% (5.2%)	Different: 71.6% (5.6%)
Invisible faces	Same: 58.2% (5.5%)	Same: 59.7% (4.1%)	Same: 64.1% (4.1%)
Task: faces discrimination	Different: 42.8% (6.8%)	Different: 46.8% (5.4%)	Different: 37.9% (5.3%)
Visible faces	Same: 79.4% (3.3%)	Same: 88.7% (2.6%)	Same: 84.8% (3.7%)
Task: eyes discrimination	Different: 27.7% (5.8%)	Different: 34.5% (7.2%)	Different: 42% (8.2%)

**Table 2:** Experiment 2: percent of trials answered the “same” when faces were “same” or “different”. In the other words, the values denote the percentages of the same answers binned for the type of stimuli (same or different faces). Columns represent eyes position (intact or shifted) and rows represent task/visibility manipulation and the time of testing. Values in parentheses denote standard error of the mean.

	Intact eyes	Shifted eyes
Invisible faces, before learning	Same: 56.6% (5.1%)	Same: 61.2% (4.2%)
Task: eyes discrimination	Different: 58.7% (5.4%)	Different: 66.2% (4%)
Invisible faces, after learning	Same: 67.9% (3.3%)	Same: 73.3% (4.7%)
Task: eyes discrimination	Different: 57.5% (4.7%)	Different: 62.1% (4.3%)
Invisible faces, before learning	Same: 45.2% (4%)	Same: 48.7% (2.9%)
Task: faces discrimination	Different: 55.2% (4.5%)	Different: 51.9% (4%)
Invisible faces, after learning	Same: 51.6% (2.8%)	Same: 54.2% (3.9%)
Task: faces discrimination	Different: 50.8% (3.5%)	Different: 49.2% (2.7%)
Visible faces, end of experiment	Same: 79.6% (3.7%)	Same: 72.5% (4.8%)
Task: eyes discrimination	Different: 31.3% (4.4%)	Different: 69.6% (4.6%)
